# Metabolomics Analysis of Litchi Leaves during Floral Induction Reveals Metabolic Improvement by Stem Girdling

**DOI:** 10.3390/molecules26134048

**Published:** 2021-07-02

**Authors:** Zuanxian Su, Qiushen Xiao, Jiyuan Shen, Houbin Chen, Shijuan Yan, Wenjie Huang

**Affiliations:** 1 Guangdong Litchi Engineering Research Center, College of Horticulture, South China Agricultural University, Guangzhou 510642, China; zuanxsu@scau.edu.cn (Z.S.); xqsycu@163.com (Q.X.); jyshen@scau.edu.cn (J.S.); 2 Agro-Biological Gene Research Center, Guangdong Academy of Agricultural Sciences, Guangzhou 510640, China; shijuan@agrogene.ac.cn (S.Y.); huangwenjie@agrogene.ac.cn (W.H.)

**Keywords:** stem girdling, flowering, young leaves, metabolic profiling, *Litchi chinensis* Sonn

## Abstract

Prolonged exposure to cold temperatures often results in a relatively low flowering rate in litchi (*Litchi chinensis* Sonn.) trees with younger leaves. This study aimed to verify the impact of stem girdling on litchi flowering by identifying and characterizing the induced metabolic changes. After a 60 day exposure to cold treatment at 15 °C/10 °C (12 h/12 h), the flowering rate of the girdled trees was 100%, while that of the non-girdled trees was 20%, indicating that girdling improved litchi flowering at its turning stage. The metabolic profiles of litchi leaves with and without stem girdling during floral induction were compared and 505 metabolites potentially associated with litchi flowering were detected. Most metabolites were involved in the metabolism of starch and sucrose, fatty acid, and phenylpyruvic acid. The metabolic pathways concerned with the biosynthesis of epinephrine, sucrose, and d-maltose were induced in leaves after girdling treatment. The level of galactitol, phenylpyruvic acid, acetyl-CoA, linoleic acid, alpha-linolenic acid, and 13-HPOT biosynthesis remained stable in the leaves from girdled trees but changed drastically in the leaves from non-girdled trees. In addition, 379 metabolites concerning flowering rate were characterized. Metabolism pathways of starch and sucrose, galactose, and linoleic acid are of great significance to the flowering of litchi. Linoleic acid exhibited the most significant variations between girdled trees and non-girdled trees with fold changes of up to 13.62. These results contribute to understanding the biological mechanism of litchi floral induction and the metabolic changes after stem girdling.

## 1. Introduction

Litchi (*Litchi chinensis* Sonn.) of the Sapindaceae family is a subtropical evergreen fruit tree. Poor or unreliable flowering can cause fluctuation in litchi production. Therefore, the complexity of litchi flowering has always been the subject of detailed studies [1,2,3,4,5,6,7]. Temperature, water stress, and the age of the shoot are critical factors affecting floral induction. A mature and terminal flush is substantial for litchi flowering [8]. A terminal flush at the stage of turning green is a vegetative organ that would compete with reproductive organs, resulting in poor flowering and alternate bearing [9]. Temperature determines whether the new buds develop into leaves or flowers [10]. Moreover, a chilling period is essential for litchi floral induction [11,12,13]. Drought can enhance litchi flowering at low temperatures as well [14,15]. However, when the cold front arrives before floral induction, the terminal shoots may be at different stages in the litchi trees, resulting in the deviation of flowering performance. Litchi growers tend to apply excessive plant growth regulators such as paclobutrazol to maintain the terminal flushes at a mature stage, which will possibly cause phytotoxicity and environmental pollution.

Physical methods such as stem girdling are widely adopted to improve plant flowering [16,17,18]. Girdling involves removing a piece of bark from the trunk or main branch of the fruit tree, thereby blocking the downward transportation of photosynthetic products and metabolites through the phloem. The timing of girdling is generally from December to February when the terminal leaves are mature enough to inhibit the sprouting of new shoots. Nevertheless, the strict demand for shoot maturity limits the application of girdling. Therefore, this study was conducted to assess whether stem girdling applied on terminal flush at the light green stage could improve floral induction.

Several studies have reported on the physiological changes in girdled plants during floral induction. Girdling intervenes in the phloem transport between roots and canopy to regulate the partitioning of photosynthates, stem CO_2_ efflux [19], and phytohormones, bringing an accumulation of carbohydrate above the girdling area [20,21,22,23,24,25]. Meanwhile, the functionality and integrity of the xylem remain unaffected by girdling [26]. The utilization of the starch reserved in roots increases after girdling [22,27]. In *Alhagi sparsifolia*, the girdling and leaf removal treatment increased the content of soluble sugars, starch, and ABA in leaves and accelerated leaf senescence [28,29]. The content of cytokinin and gibberellin in branches has also been altered by girdling [30]. Starch levels slightly increase in trees with branches girdled and de-fruited [31]. Accumulation of carbohydrates produces short-term feedback inhibition of photosynthesis and long-term gene expression modifications associated with carboxylase oxygenase, leaf-specific ADP glucose pyrophosphorylase, and anthocyanin pathway. Although several studies have reported impressive results on girdling, the metabolic changes after girdling are still not clear and making sense of the changes is necessary because metabolites are the final response to genetic and environmental changes [32]. Identifying and characterizing the metabolic profile of litchi leaves after girdling and during floral induction will enrich the understanding of girdling and flowering mechanisms in litchi.

In this study, stem girdling and cold treatment were applied to potted litchi trees with terminal flushes at the light green stage. Ultra-high performance liquid chromatography-quadrupole time-of-flight mass spectrometry (UPLCQ-TOF/MS) was adopted to obtain leaf metabolites during floral induction. In addition, the metabolite profiles were characterized to identify potential metabolites in response to girdling and cold. The results are expected to provide strong evidence for improving litchi floral induction and a solid foundation for further research on functional metabolites in litchi.

## 2. Results

### 2.1. Biological Presentation of Litchi Trees under Experimental Conditions

**Figure 1:**
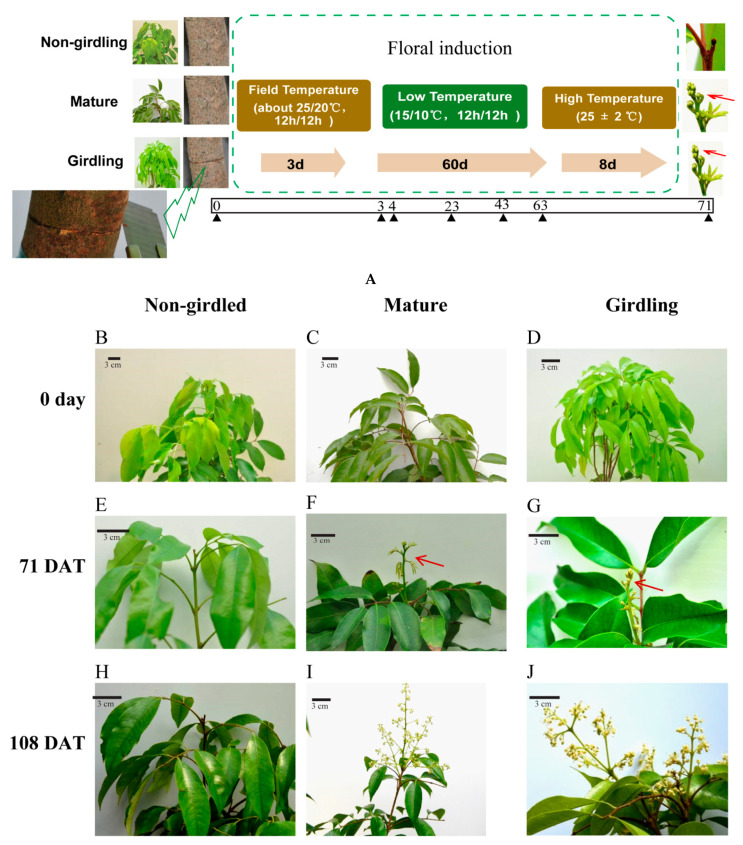
Experimental schematic and the effect of stem girdling on litchi flowering. (**A**): Schematic of the experimental treatments. The black triangles indicate the time of sampling. (**B**–**J**): Non-girdled, mature, and girdled Litchi trees displayed variation in flowering after floral induction. (**B**–**D**): Representative photographs of the terminal flush on trees before any treatment on 0 day. (**E**–**G**): Representative photographs of trees after floral induction. (**E**,**H**): Representative photographs of non-girdled trees that did not form inflorescences primordium. (**F**,**G**): Representative photographs of trees formed inflorescences primordium. The red arrows indicate inflorescences primordium. (**I**,**J**): Representative photographs of trees bloomed. DAT is the abbreviation of days after treatment. The apical leaves of the terminal flushes in girdling trees and non-girdled trees were expanded to full size completely and displayed light green. The apical leaves of the terminal flushed in mature trees were fully mature.

All girdled and mature trees bloomed, while only 20% of the non-girdled trees flowered (Figure 1 and Table 1). The percentage of flowering shoots of girdled trees (71.97% ± 10.06%) was close to that of mature trees (74.23% ± 17.93%) and significantly higher than that of the non-girdled trees in the control group (10.08% ± 9.14%). Flowering in girdled trees initiated 3.5 days earlier than non-girdled trees. The floral differentiation days, panicle length, and panicle width varied between girdling and non-girdling trees.

**Figure 2:**
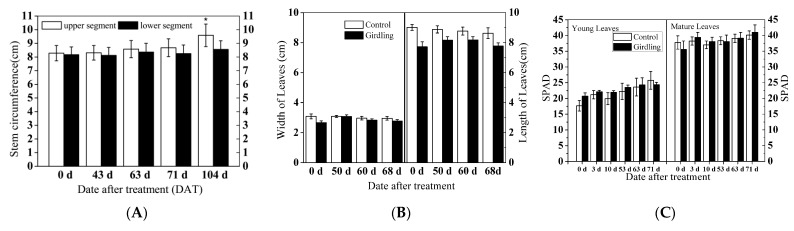
The growth of litchi trees was suppressed by cold treatment. (**A**): Variations between the upper and lower segments of the girdle. Asterisks (*) indicate significant differences determined by an unpaired *t*-test at *p* < 0.05. (**B**): Length and width of leaves in the terminal shoots. (**C**): Measurement of SPAD value of the leaves on the terminal flushes. SPAD, value of Soil and Plant Analyzer Development, represented Chlorophyll content.

The growth rate of the upper segment has not increased as significantly as the lower segment during floral induction. Few girdled or non-girdled trees grew during floral induction according to the SPAD values, length, and width of leaves in the terminal shoots (Figure 2).

### 2.2. Correlation Coefficient of Metabolite Profiles in Response to Stem Girdling and Low Temperatures

**Figure 3:**
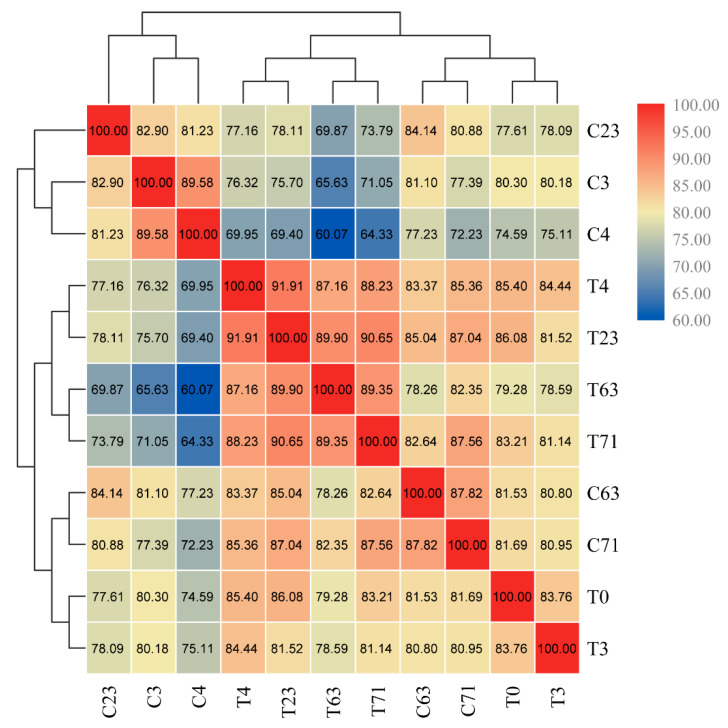
Spearman’s rank correlation coefficient of all metabolite profiles from samples collected at all sampling time. C indicates samples from trees in the control group. T indicates samples from trees in the girdled group. The numbers 0, 3, 4, 23, 63, and 71 indicate the sampling time.

Spearman’s rank correlation coefficients were calculated between samples and presented in a heat map (Figure 3). All samples were classified into three clusters. Cluster one contained samples in the control group at low temperatures for 0 day (C3), 1 day (C4), and 20 days (C23). Cluster two included samples in the girdled group at low temperatures for 1 day (T4), 20 days (T23), and 60 days (T63) and then at high temperatures for 8 days (T71). Cluster three involved samples before girdling (T0), 3 days after girdling (T3), control samples at low temperature for 60 days (C63), and control samples for 71 days (C71).

The samples with girdling were categorized into two groups. T4, T23, T63, and T71 were in one group, while T0 and T3 were sorted into another group. The group categorization suggested that low temperatures could cause significant metabolite changes in girdled trees on T4. Notably, the Spearman’s rank correlation coefficient of T4 and T3 is lower than that of C4 and C3, meaning that the metabolite difference between T4 and T3 is enlarged even though T4 and C4 trees experienced low temperatures for only 1 day. Similarly, the Spearman’s rank correlation coefficient of T63 and T3 is lower than that of C63 and C3, indicating that the metabolite difference of girdling is greater than that in the control group during floral induction. These results indicate that integrative effects of girdling and low temperatures are required to induce particular metabolites. Therefore, metabolites that responded to girdling, low temperatures, and their combination should be characterized.

### 2.3. PCA of Metabolic Changes

The metabolites of samples at 0, 3, 4, 23, 63 days, and 71 DAT were compared with non-targeted UPLC-QTOF/MS and a total of 22,251 compounds were detected. PCA with quality control (QC) samples was carried out to estimate the achievement of the UPLC-QTOF/MS approach. The data shows that all the QC samples are clustered together, suggesting that the approach has good reproducibility.

**Figure 4:**
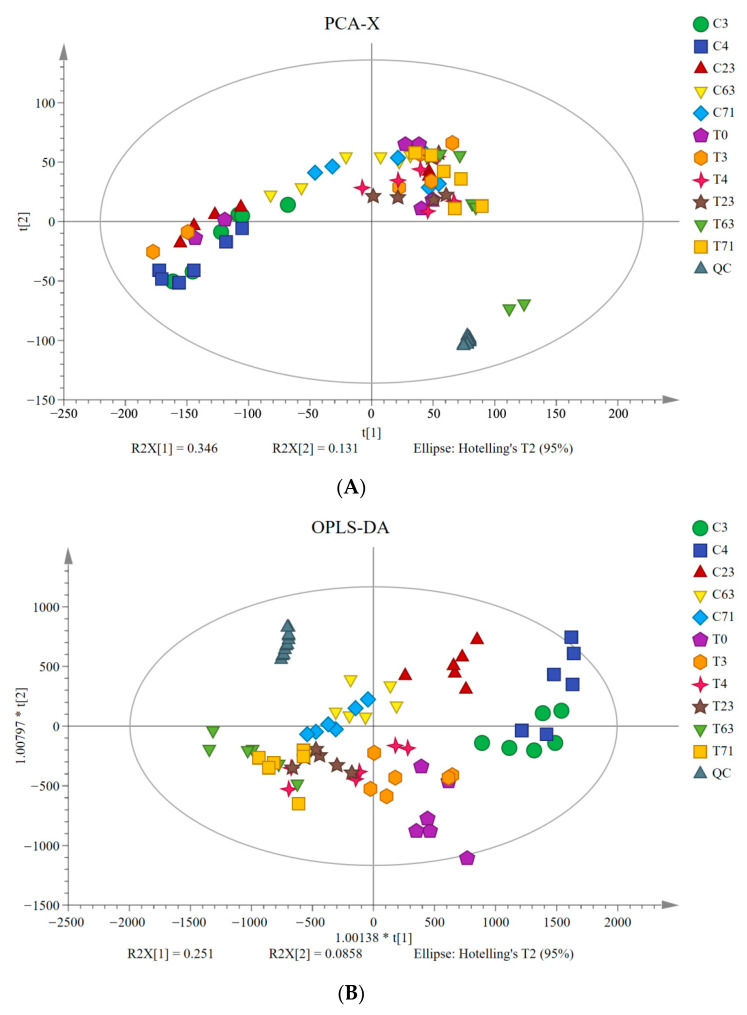
Principal component analysis (PCA) score plots and orthogonal projections to latent structures-discriminate analysis (OPLS-DA) score plots showed the metabolomics trajectory of picked “Guiwei” litchi leaves in girdling group and control group under floral induction. (**A**): The PCA score plot of all metabolites from the six biological replicates of the litchi leaves collected at all sampling time. Scores were plotted with principal component 1 in the *x*-axis and principal component 2 in the *y*-axis. (**B**): OPLS-DA score plot of all metabolites from the six biological replicates of the litchi leaves collected at all sampling time. QC indicates the quality control sample.

According to the PCA results, the leaf samples of non-girdled trees are separated from those of the girdled trees. The first two principal components can explain 47.7% of the variation in metabolites. The first principal component (PCA1) and second principal component (PCA2) represent 34.6% and 13.1% of the total variation. PCA1 clearly distinguishes C3 from T3 and C4 from T4, but it could not distinguish other treatments. PCA2 separates the plots of C23 and T23. Results show a vital difference between the girdled and non-girdled trees (Figure 4A).

In order to screen the orthogonal variables in the metabolites not related to the categories and to obtain more reliable and different metabolites information among groups, the orthogonal projections to latent structures-discriminate analysis (OPLS-DA) was conducted to screen the maximum metabolites information between non-girdled and girdled trees. The scatter plots (Figure 4B and Figure S1) show an evident separation between girdled and non-girdled trees. Moreover, these models are interpreted by high values of R2Y. The diagrams suggest a sound model quality in the modeling analysis of OPLS-DA. The leaf metabolites are significantly different between girdled and non-girdled trees.

The variable importance in projection (VIP, >1) and Student’s *t-*test (*p* < 0.05) were adopted as a criterion to observe differential metabolites. A total of 817 differential metabolites were distinguished in compliance with the public database (http://www.hmdb.ca/; http://www.lipidmaps.org/, accessed on 16 October 2017) and a self-built database.

### 2.4. Global View of Metabolites Associated with Flowering 

**Figure 5:**
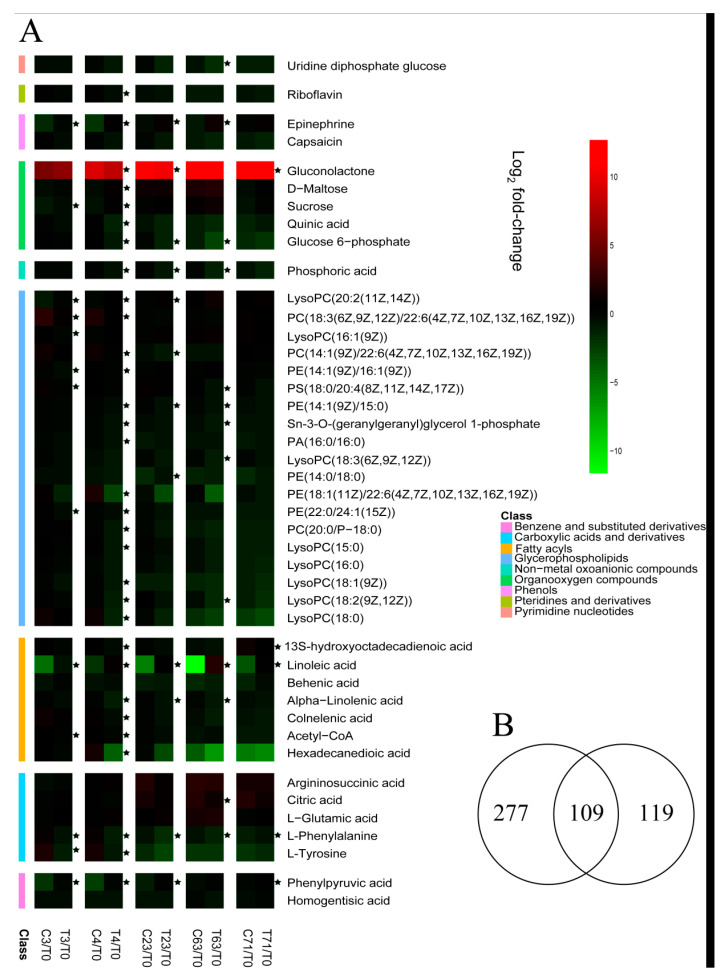
Metabolites accounting for flowering in Guiwei litchi leaves from terminal flush at the turning stage were screened. (**A**): Heat map of fold-changes relative to 0 day for metabolites accounting for flowering in Guiwei litchi leaves from terminal flushes. C indicates samples of trees from the control group. T indicates samples of trees from the girdled group. The numbers 0, 3, 4, 23, 63, and 71 indicate the sampling time. Values represent the means of log2-transformed fold-changes from six biological replicates relative to the T0 (before treatment). Asterisks (*) indicate significant differences between treatments determined by an unpaired *t*-test at *p* < 0.05. (**B**): Venny diagram of group comparison for metabolic profiles for flowering in the control group and the girdled group. C3 vs. C63 indicates different metabolites in Guiwei litchi leaves in the control group during floral induction. T3 vs. T63 indicates different metabolites in Guiwei litchi leaves in the girdled group during floral induction. A total of 505 metabolites were identified in this study and the numbers in the figures indicate the numbers of different metabolites for each comparison.

After exposure to low temperatures, girdled and non-girdled litchi trees initiated 60 days according to the Venny diagram that showed pairwise comparisons of the differential metabolites in the C3, C63, T3, and T63 samples. A total of 505 differential metabolites appeared after 60 days (Figure 5B, Supplementary Material), which might be potentially be associated with litchi floral induction. The metabolites are chiefly related to carbohydrates, stress response, energy metabolism, secondary metabolism, protein degradation, and small molecular compounds such as organic acids and amino acids. According to the KEGG database, 22 enriched metabolic pathways were found (Figure S2). The responses of the metabolites were also detected by computing the fold-changes relative to T0 day (Figure 5A) to analyze the metabolic changes. Metabolites are divided into nine groups: benzene and substituted derivatives, carboxylic acids and derivative, fatty acyls, glycerophospholipids, non-metal oxoanionic compounds, organooxygen compounds, phenols, pteridines, and derivative sand pyrimidine nucleotides.

**Figure 6:**
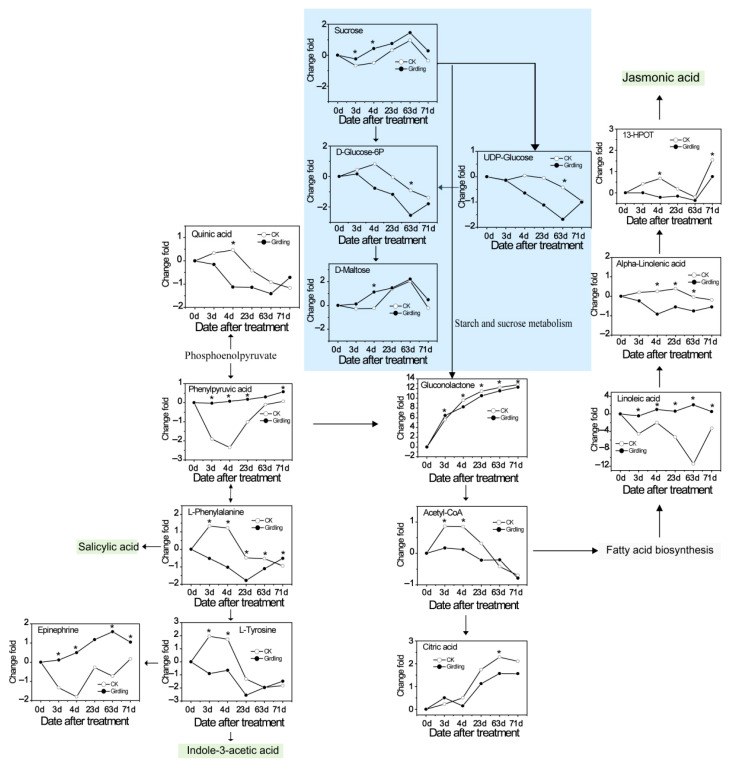
Changes of metabolite concerning flowering in metabolic pathways in Guiwei litchi leaves of terminal flush. CK indicates samples of trees from the control group. Girdling indicates samples of trees from the girdled group. The numbers 0, 3, 4, 23, 63, and 71 indicate the sampling time. Values represent means of log_2_-transformed fold-changes from the six biological replicates relative to 0 day. Asterisks and five-pointed stars indicate significant differences between the girdled and control groups determined by an unpaired *t*-test at *p* < 0.05. White dots represent samples of trees from the control group. Black dots represent samples of trees from the girdled group.

The remarkable changes of metabolites concerning flowering in litchi leaves from the girdled group compared with the control group are shown in Figure 6. Most metabolites are involved in starch and sucrose metabolism, fatty acid metabolism, and phenylpyruvic acid metabolism. The accumulations of epinephrine, sucrose, and d-maltose are induced in leaves from the girdled group. However, the d-glucose-6P, UDP-glucose, and quinic acid biosynthesis levels are relatively low in leaves from the non-girdled and girdled trees. Furthermore, the galactitol, phenylpyruvic acid, acetyl-CoA, linoleic acid, alpha-linolenic acid, and 13-HPOT biosynthesis contents remain stable in leaves from the girdled trees but are low in leaves from the control group. Linoleic acid exhibited the most significant variations between girdled trees and non-girdled trees with fold-changes of up to 13.62 (Figure 6).

The contents of carbohydrates and IAA are measured during floral induction. The girdled trees display a significantly faster rise in glucose and sucrose on 3 DAT and 4 DAT than the non-girdled trees. It is noteworthy that girdled trees show a higher starch content than non-girdled trees from 23 DAT to 63 DAT. The level of IAA in the girdled trees is lower than that in the non-girdled trees on 23 DAT and 43 DAT (Figure S4). These results indicate that girdling might decelerate the utilization or the export of assimilates, thus increasing the soluble sugars at an early stage and promoting starch accumulation at a later stage. Meanwhile, girdling can suppress the synthesis pathway of IAA.

### 2.5. Metabolites Accounting for Different Flowering Rate

**Figure 7:**
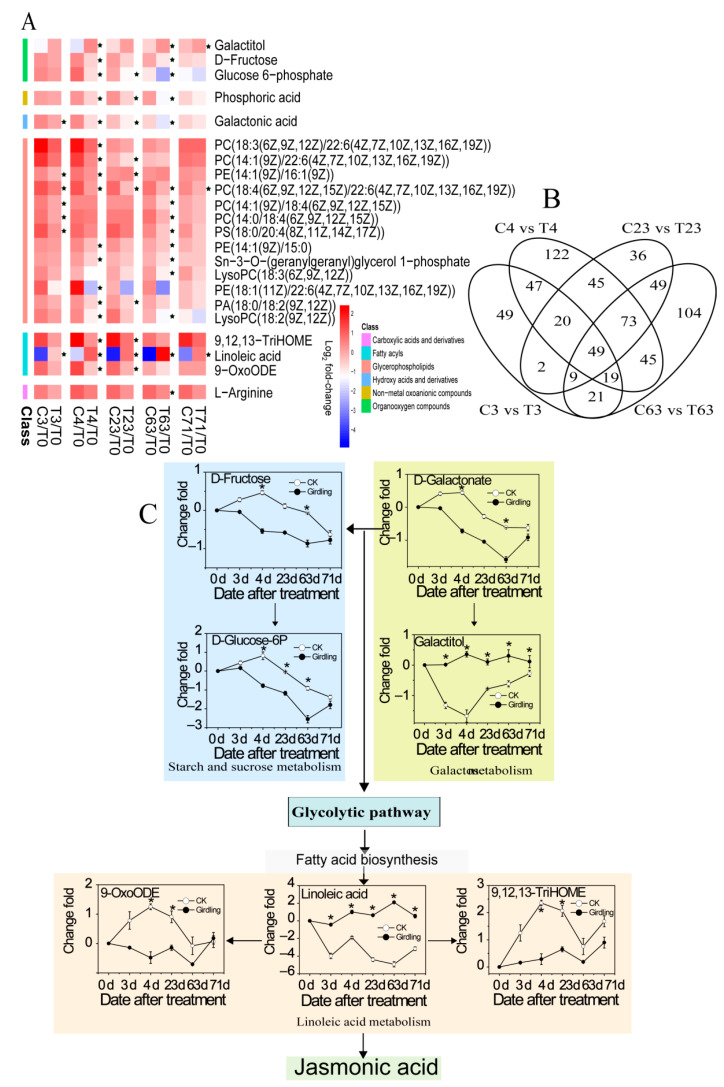
Metabolites accounting for flowering rate in Guiwei litchi leaves from terminal flushes at turning stage were screened. (**A**): Heat map of fold-changes relative to 0 day for metabolites accounting for flowering rate in Guiwei litchi leaves from terminal flushes. C indicates samples of trees from the control group. T indicates samples of trees from the girdled group. The numbers 0, 3, 4, 23, 63, and 71 indicate the sampling time. Values represent the means of log2-transformed fold-changes from six biological replicates relative to the T0 (before treatment). Asterisks (*) indicate significant differences between treatments determined by an unpaired *t*-test at *p* < 0.05. (**B**): Venny diagram of group comparison for metabolic profiles accounting for flowering rate in the control and girdled groups. C3 vs. T3 indicates different metabolites in Guiwei litchi leaves in the comparison between the control and girdled groups at 3 DAT. C23 vs. T23 indicates different metabolites in Guiwei litchi leaves in the comparison between the control and girdled groups at 23 DAT. C63 vs. T63 indicates different metabolites in Guiwei litchi leaves in the comparison between the control and girdled group at 63 DAT. A total of 379 metabolites that emerged at least twice in two comparisons were identified and the numbers in the figures indicated the numbers of different metabolites for each comparison. (**C**): Changes of metabolites accounting for flowering rate in metabolic pathways in Guiwei litchi leaves of terminal flush. CK indicates samples from trees in the control group. Girdling indicates samples from trees in the girdled group. Numbers 0, 3, 4, 23, 63, and 71 indicate the sampling time. Values represent means of log_2_-transformed fold-changes from the six biological replicates relative to 0 day. Asterisks and five-pointed stars indicate significant differences between the girdled and non-girdled trees determined by an unpaired *t*-test at *p* < 0.05. White dots represent samples of trees from the control group. Black dots represent samples of trees from the girdled group.

All girdled trees flowered while only one non-girdled tree did. The percentage of flowering shoots of girdled trees is remarkably higher than that of non-girdled trees. The metabolomic profiles of leaves at the turning stage are analyzed in line with the pairwise comparison to identify the metabolites accounting for different flowering rates. A total of 815 differential metabolites appeared (Figure 7B), 379 of which emerged at least twice in two comparisons of the low-temperature treatment (Supplementary Material). Therefore, the KEGG annotation and the primary enrichment metabolic pathways of these potential candidate metabolites need to be considered. The metabolites are mainly related to carbohydrates, stress response, energy metabolism, and protein degradation (Figure S3). The responses of the metabolites in nine enrichment pathways were measured by computing the fold-change relative to T0 day (Figure 7A) to analyze the metabolic changes accounting for the different flowering rates between the control and girdled groups. The metabolites are divided into six groups: organooxygen compounds, hydroxy acids and derivatives, glycerophospholipids, fatty acyls, carboxylic acids and derivatives, and non-metal oxoanionic compounds.

The outstanding metabolite changes in leaves associated with different flowering rates were analyzed. Moreover, d-galactonate, d-fructose, and d-glucose-6P biosynthesis are inhibited in leaves from the girdled and non-girdled trees, while the biosynthesis of galactitol, linoleic acid, 9,12,13-triHOME, and 9-oxoODE remain stable in leaves from the girdled group but vary in leaves from the control group. Linoleic acid exhibited the most significant variations between girdled trees and non-girdled trees, with fold changes of up to 7.02 (Figure 7C).

## 3. Discussion

### 3.1. Stem Girdling Promotes Litchi Flowering

Our previous results showed that after the exposure to 60 days of cold treatment, the trees with terminal flushed during the light green stage (before maturity) did not flower, while trees with terminal flushed during the mature stage fully flowered [6]. In this research, potted litchi trees with terminal flushed at the light green stage or mature stage were applied to evaluate the effect of stem girdling on flowering. The pre-girdled trees were capable of flowering similar to mature trees, while the non-girdled trees had a shallow flower rate after exposure to a 60 days cold treatment (Figure 1 and Table 1). Furthermore, girdled trees flowered earlier than non-girdled trees. The above results suggested that pre-treatment of stem girdling before low temperature could improve the flowering of litchi, even though the terminal flush was at a light green stage. 

The relative chlorophyll content was measured to observe leaf maturation [33]. In this study, chlorophyll content represented by SAPD values remained stable during the induction period, which indicates that low temperatures probably inhibited leaf maturation and does not influence the physiological shifts to flower initiation.

### 3.2. Stem Girdling Alters the Carbohydrate Metabolites during Floral Induction

Several metabolites in Starch and sucrose metabolism are affected by stem girdling. Sucrose and d-maltose in leaves of girdled trees increase significantly in concentration by 0.92-fold and 1.37-fold at 4 DAT, while Glucose 6p and UDP-Glucose decreased by 1.64-fold and 1.26-fold at 63 DAT (Figure 5A and Figure 6). Carbohydrates are particularly relevant to physiology in metabolites transported through plant cell membranes because carbohydrates act as fuels for energy metabolism and provide precursors in many anabolic reactions [34]. Moreover, sugar can affect the onset of flowering [35], regulate flowering time [36], and contribute to biotic as well as abiotic stress tolerance [37,38]. Transitory starch and soluble sugars can promote flowering by serving as signals and energy resources [39,40,41]. Sucrose is synthesized by photosynthetically fixed carbon, starch, or lipid in the cytosol, a significant transport metabolite in long-distance carbon transport between sinks and sources. The increase in sucrose export is related to starch catabolism [42]. The early increase in sucrose content does not appear to come from increased photosynthesis but from carbohydrates (possibly starch) stored in the stems and leaves [43,44], which is consistent with previous studies on the contents of sucrose and starch on 0 DAT and 3 DAT (Figure S3). Moreover, a high level of sucrose can amplify the expression of flowering-related gens such as *CO*, *FT* [45], and *LFY* [46]. Considering the enormous amount of sucrose and d-maltose, as well as the consumption of UDP-glucose and glucose 6-phosphate, starch and sucrose metabolism are probably essential for litchi floral induction. A high level of sucrose, d-maltose, and starch in the leaves of girdled trees during the chill condition can improve cold stress tolerance and provide plenty of energy for morphological floral differentiation.

The level of gluconolactone in girdled trees decreases by 0.54-fold to 1.35-fold at 4 DAT to 71 DAT than that of non-girdled plats (Figure 5A and Figure 6). Gluconolactone is converted from glucose via glucose oxidase. Gluconolactone polymerizes with aminopropyl methacrylamide to form a continuous expansion-strengthened polymethacrylamide, correct injuries through self-repair mechanisms, and increase resistance to acute mechanical stress [47]. Gluconolactone is the precursor of gluconolactone-6-phosphate in the pentose phosphate pathway, which is usually accumulated in insects when insects are exposed to harsh environmental conditions [48]. Low temperatures for floral induction are also a stress condition resulting in gluconolactone in litchi leaves. Moreover, δ-gluconolactone is an inhibitor of trehalase, which is involved in flowering [49]. Therefore, stem girdling probably reduces the accumulation of gluconolactone and the sensitivity of young leaves to low-temperature. Furthermore, gluconolactone is an adverse factor for flowers possibly.

### 3.3. Stem Girdling Effect on the Phosphoenolpyruvate Metabolites during Floral Induction

Phenylalanine metabolites are regulated by stem girdling. The amount of phosphoenolpyruvate metabolites, such as the amount of phenylpyruvic acid, l-phenylalanine, and l-tyrosine, is remarkably different during the experiment. The level of phenylpyruvic acid in leaves of the control group decreases by 1.88-fold at high temperatures (3 DAT) and by 2.4-fold in the early stage of the chilling condition (4 DAT) and then keeps increasing until the end of treatment while the level remains stable. The contents of l-phenylalanine and l-tyrosine in leaves of non-girdled trees are high at room temperature (0 DAT) and then decreases dramatically by 0.56-fold to 2.25-fold in cold conditions, while the level of that in girdled trees keeps decreasing until 23 DAT and then increases until the end of the experiment (Figure 5A and Figure 6). Trees have higher demands for aromatic amino acids such as l-phenylalanine and l-tyrosine, which perform as precursors of many aromatic primary and secondary metabolites [50]. Phenylalanine metabolism is diverse and prevalent in trees. About 20–30% of photosynthetic carbon can be used to produce phenylalanine production [51] and phenylalanine-derived compounds, constituting about 30–45% of plant organic substance [52] and incurring significant impacts on growth and development [53], reproduction [54], and defense [55,56]. Aromatic amino acids protect trees from abiotic stresses, such as high temperature [57,58]. The dynamic change of phenylalanine metabolites indicates that they can increase during the early stages of development and decrease or remain relatively constant after that [59,60]. l-tyrosine is a precursor for IAA, which is a flowering inhibitor in litchi [61]. The pattern of changes in IAA levels (Figure S4) is consistent with l-tyrosine. Girdling probably reduces the phenyl pyruvic acid catabolism that can produce l-phenylalanine and l-tyrosine, sequentially inhibiting IAA’s level with a high percentage of flowering rate. 

### 3.4. Stem Girdling Effect on the Catecholamine Metabolites during Floral Induction

Epinephrine, one of the catecholamines, can increase the level of cyclic adenosine monophosphate (cAMP). The accumulation of epinephrine in girdled trees increases by 2.30-fold and 2.31-fold at 3 DAT and 63 DAT than in control trees, respectively (Figure 5A and Figure 6). Due to limited research, epinephrine has not been considered to be a targeted metabolite involved in girdling and litchi flowering. However, many studies have shown that epinephrine and its analogue are crucial in the flowering. First, epinephrine can partly overcome the inhibition posed by sucrose-ammonium of flowering in [62] *L.paucicostata* [62,63]. Second, the antagonist of epinephrine and propranolol can inhibit the flowering of *Lemna* with an ammonium-free medium. The epinephrine in the flowering of the plant should be relevant to the alteration of intracellular levels of cAMP or cCMP-like compounds [63]. Third, catecholamines are probably involved in *pharbits nil* floral induction. Inhibitors of catecholamine biosynthesis can inhibit floral induction in *Pharbits nil* as well. Furthermore, the floral induction can be recovered when norepinephrine are exogenously fed with inhibitors of catecholamine biosynthesis [64]. Fourth, l-epinephrine, l-isoproterenol, l-norepinephrine, and their reaction products with (12*Z*, 15*Z*)-9-hydroxy-10-oxo-octadeca-12,15-dienoic acid (KODA) can substantially promote flowering in *Lemna paucicostata* [65,66] by inducing floral primordial and improving flower development [67]. Finally, catecholamine with tricyclic structure originated from the conjugation of fatty shows flowering activity [68]. Therefore, the girdling probably promotes epinephrine biosynthesis, which is involved in the girdling and flowering in litchi. However, further investigation is required to clarify this speculation.

### 3.5. Stem Girdling Effect on the Acetyl-CoA Amount during Floral Induction

The level of acetyl-CoA in girdled trees is more stable than that of non-girdled trees, even though the level of acetyl-CoA decreases during floral induction regardless of what treatments they received (Figure 5A and Figure 6). Acetyl-CoA is activated by acetyl-CoA carboxylase (ACCase) to form malonyl-CoA. Acetyl-CoA is applied in plastid for fatty acid synthesis and is essential for fatty acid elongation [69]. Meanwhile, the acyl-CoA oxidase catalyzes the β-oxidation pathway. The mutant of *Arabidopsis 3-ketoacyl-CoA thiolase-2* (*kat2-1*) can inhibit the increased flowering and reduce reproductive success, showing that a functional acyl-CoA and β-oxidation pathway is required to maintain the balance between the initiation of floral meristems and the silique development [70]. Moreover, the reduced levels of *AACT2* show that CoA thiolase genes in *Arabidopsis* (*AACT*) can elongate flowering duration [71]. Therefore, girdling probably inhibits the acetyl-CoA biosynthesis or promotes its catabolism, which might play an indirect role in litchi floral induction.

### 3.6. Stem Girdling Effect on the Fatty Acid Metabolites and Catabolism during Floral Induction

The changing patterns of metabolites and fatty acid catabolism of non-girdled trees are remarkably different from girdled trees. The amount of linoleic acid in leaves in non-girdled trees decreases by 13.62-fold at 64 DAT than that of girdled trees. The amount of linoleic acid in leaves of girdled trees remains stable, while the content in non-girdled trees decreases dramatically at high temperature (3 DAT) and chill condition (4 DAT to 63 DAT) and then increases until the end of treatment. The levels of alpha-inolenic acid and 13S-hydroxyoctadecadienoic acid (13-HPOT) in leaves of girdled trees are lower than those of the non-girdled trees (Figure 5A and Figure 6). Oxylipins are a big family of lipid-derivatived metabolites that are crucial in plant defense. These compounds are originated from initial oxidation fatty acids, mostly linolenic (18:3) and linoleic acids (18:2) [72,73,74,75]. Among the oxylipins, JA, 13-LOX derivatives, α-DOX, and 9-DOX participate in plant defense and development by acting as signals that regulated gene expression [76,77,78,79]. Moreover, 13-LOX is induced in leaves for mechanical damage [80]. Furthermore, 13-hydroperoxides probably serve as substrates for alternative allene oxide synthase, resulting in the formation of jasmonic acid, which accumulates upon wounding and activates defense-gene expression [81]. The participation of the linoleic acid, α-linoleic acid, and 13-HOPT show that stem girdling probably decreases the sensitivity of immature leaves to the low-temperature stress and reduces the accumulation of metabolites of fatty acid catabolism, which is possibly an adverse factor for flowering. However, whether the oxylipin pathway plays a role in litchi flowering or not needs to be further investigated.

### 3.7. The Accumulation of Metabolites Accounting for Different Flowering Rate 

Figure 7A,C illustrate the accumulation of metabolites accounting for a different flowering rate between non-girdled trees and girdled trees. The levels of d-galactonate, d-fructose, and d-glucose-6P in leaves of girdled trees are lower than those in non-girdled trees. The amount of galactitol in the leaves of non-girdled trees decreases by 2.3-fold at 4 DAT than that of girdled trees. The amount of galactitol in leaves of girdled trees remains stable while the level of galactitol in non-girdled decreases dramatically at room temperature (3 DAT) and the early stage of the chilling condition (4 DAT) and then it proceeds to keep increasing until the end of treatment. The amount of linoleic acid metabolites such as linoleic acid, 9, 12, 13-TriHOME, and 9-OxoODE in the leaves of girdled trees remains stable. The level of linoleic acid in the leaves of non-girdled trees decreases (2.9-fold to 7.02-fold) dramatically, while the levels of 9, 12, 13-TriHOME, and 9-OxoODE increase accordingly. Moreover, 9-OxoODE, 13-OxoODE, as well as 9-HODE and 13-HODE can be released in response to noxious thermal stimuli [82]. The results suggest that starch and sucrose, galactose, and linoleic acid might play a crucial role in the flowering of litchi. It seems that girdling probably reduce the susceptibility of leaves at the light green stage, resulting in relatively stable levels of d-fructose, d-glucose-6P, d-Galactonate, galactitol, linoleic acid, 9-OxoODE, and 9, 12, 13-triHOME during floral induction, which could possibly increase the flowering rate. However, further investigation is needed to prove this hypothesis.

## 4. Materials and Methods

### 4.1. Plant Materials and Experimental Scheme

Six year old air-laying potted litchi (*Litchi chinenesis* cv. Guiwei) trees were planted in 20 L pots with coconut chaff, mushroom, and loam (*v*:*v*:*v* = 1:1:3). Trees about 1.5 min height were selected for the experiment. These trees were divided into two groups according to the stage of terminal flushes. The trees with apical leaves of the terminal flushes, which were expanded to full size completely and displayed light green, were assigned as trees that were turning green. The trees with the apical leaves that flushed fully mature were designated as mature trees. Six mature trees and twelve turning-green trees receive treatments, as shown in Figure 1A. 

Six turning green trees were girdled (removing a 0.1 cm wide ring of bark and cambium) with a utility knife. The ring was about 20 cm from the base of the tree stem. The mature trees and the rest of turning-green trees were considered as a control group. All trees were placed in a plastic greenhouse with the temperature conditioned at about 25/20 °C (day/night). Three days later, all trees were transferred into a cold plastic greenhouse and adequately watered every two days at low temperatures (15/10 °C, 12/12 h), relative humidity of 75–80%, and full sunlight (The normal daylight photosynthetic photon flux density between the hours of 12:00 p.m. and 13:00 p.m. was between 300 and 1400 μmol m^−2^ s^−1^. Regular lights were supplemented between the hours of 07:00 a.m. and 19:00 p.m. to support natural sunlight and to minimize shading. The photosynthetic photon flux density of regular lights was 160 μmol m^−2^ s^−1^). The trees were moved around every 3 days to avoid a possible uneven cold treatment. After sixty days, the temperature of the greenhouse was switched to 25 ± 2 °C with the same humidity, light, and water conditions until floral initiation (71 DAT). The flowering rate of trees, percentage of flowering shoots, days to floral initiation, days to male and female flowers floral differentiation, panicle length, and panicle width of each group were recorded accordingly. Floral induction days represented the period from the first day of the experiment to the day of panicle primordium emergence. Floral differentiation days denotes the period from the day panicle primordium emerged to the day male flowers or female flowers emerged. Panicle length and width were also measured on the longest and widest parts of the largest panicle in each inflorescence. 

For trees at the stage of turning green, Soil and Plant Analyzer Development (SPAD) values of apical leaves in terminal shoots were measured with portable Chlorophyll Meter Model SPAD-502 (Konica Minolta, Japan). The third to fifth compound leaves were collected at 0, 3, 4, 23, 43, 63, and 71 DAT. All leaf samples were frozen in liquid nitrogen and then stored at −80 °C. Every sample consisted of six biological replicates.

### 4.2. Metabolite Extraction

Litchi leaves were ground into a powder with a mortar and a pestle in liquid nitrogen. Samples were then extracted as specified by the instructions of Majorbio Bio-Pharm Technology Co., Ltd. (Shanghai, China) [83,84] with minor modifications. The amount of 60 mg of ground leaf samples was added to 0.6 mL methanol/water (7:3, *v*/*v*) and 20 μL of l-2-Cl-Phe (0.3 mg/mL) was used as an internal standard. After ultrasonication for 30 min, the samples were incubated for 20 min at −20 °C. Then, the extracts were centrifuged at 14,000 rpm at 4 °C for 10 min. Finally, 200 μL of supernatant was filtered through a 0.2 μm filter and then injected into a 2 mL vial for liquid chromatography/mass spectrometry (LC/MS) analysis.

### 4.3. Metabolite Profiling

The extracts were profiled using a Waters VION IMS Q-TOF Mass Spectrometer equipped with an electrospray interface (Waters Corporation, Milford, MA, USA) platform and chromatographic separation was performed on an ExionLCTM AD system (AB Sciex, Framingham, MA, USA) equipped with an AcquityTM UPLC system. The bridged ethyl hybrid (BEH) C18 column (100 mm × 2.1 mm, 1.7 µm; Waters, Milford, MA, USA) was used. The mobile phase consisted of (A) 0.1% formic acid in water and (B) 0.1% formic acid in acetonitrile. The gradient elution program was set as follows: 0–2 min: 5–20% B; 2–8 min: 20–60% B; 8–12 min: 60–100% B; 12–14 min: 100% B; 14–14.5 min:100% to 5% B; and a final 14.5–15.5 min: 5% B. The flow rate was 0.40 mL/min. The column was 40 °C. The injection volume was 3 μL. 

For MS analysis, both negative and positive ion scan modes were tested. Capillary voltage, injection voltage, and collision voltage were set to 1.0 kV, 40 V, and 6 eV, respectively. Source temperature and solvent temperatures were 120 °C and 500 °C, respectively. The solvent gas flow rate was at 900 L/h. The scan time and the interscan delay was 0.1 s and 0.02 s, respectively. The MS scan range was 50–1000 *m*/*z*.

Blank samples were injected before biological samples. All biological samples were injected in sequence. The quality control (QC) samples were made by mixing equal volumes of all samples. A QC sample was inserted for every 10 analytical samples to evaluate the stability of the analytical system and to assess the reliability of the results. 

### 4.4. Carbohydrates Determination

Soluble sugars were extracted and detected by high-performance liquid chromatography (HPLC) and Agilent 1200 HPLC system (Agilent Technologies, Waldbronn, Germany) in line with the protocol of Yang [85], with a minor modification. In addition, 0.3 g leaf samples were homogenized with a mortar and a pestle in 4 mL 90% (*v*/*v*) ethanol. The homogenate was then transferred to a 15 mL centrifuge tube. The mixture was heated in water at 80 °C for 30 min and then centrifuged at 4000 rpm for 10 min at room temperature. The centrifuge tube was centrifuged at 13,000 rpm for 10 min. The supernatant was sieved with a Sep-Pak^®^ 1cc (100 mg) C18 cartridge (Waters Corporation, Milford, MA, USA). A CARB Sep Coregel 87 C column (Transgenomic CHO-99-5860, Omaha, NE, USA) was utilized. The ultra-pure water was also used as the mobile phase at a 0.4 mL/min flow rate. The column was maintained at 80 °C. The sugars were distinguished by the retention time to those of standards (sucrose, glucose, and fructose standards, purchased from Sigma Chemical Co, St. Louis, MO, USA). The quantification of individual sugars in samples was performed with peak areas and calibration curves derived from external standards.

The precipitate was applied for starch determination. It was extracted two times with 10 mL 80% ethanol then bathed in the water at 100 °C for 30 min. The mixture was centrifuged at 4000 rpm for 10 mins with supernatant abandoned. The precipitate was then re-suspended in 5 mL 80% (*w*/*v*) calcium nitrate and bathed in boiling water for 20 min. The mixture was centrifuged at 4000 rpm for 10 min. The supernatant was transferred to a 25 mL scaled test tube and diluted to 25 mL with 80% (*w*/*v*) calcium nitrate and adopted for starch determination with the I_2_–KI method in compliance with the protocol of Xu [86]. 

### 4.5. Indole-3-Acetic Acid (IAA) Determination

IAA was extracted from the samples. First, 50 mg powder of frozen plant tissues were transferred to 2 mL screw-cap tubes. Next, 50 µL working solution with an internal standard was added to a 2 mL tube. Then, 500 µL extraction solvent was prepended to each tube. The tubes were shaken and centrifuged three times at 4 °C. The solvent mixture was concentrated with a nitrogen evaporator. Additionally, 10 μL of extracted solution from each sample was injected into the LC-MS system. Then, the eluate was adopted into the electrospray ion source of a tandem triple quadrupole MS analyzer (API4000, AB SCIEX, Foster City, CA, USA) and the IAA compounds were quantified in multiple reaction monitoring (MRM) mode. The MS conditions were as follows: source, Turbo IonSpray; ion polarity, negative; ion spray voltage, −4500 V; ion polarity, positive; ion spray voltage, 5500 V; source temperature, 550 °C; gas, nitrogen; curtain gas, 30 psi; nebulizing gas (GS1), 55 psi; Collision gas (GS2), 55 psi; scan type, MRM; Q1 resolution, unit; Q3 resolution, unit. 

### 4.6. Statistical Analysis

All metabolite profiles of samples were compared to work out the expression of the metabolites. Furthermore, the Spearman correlation between samples was calculated using the ‘cor’ function in R programming language (http://www.r-project.org, accessed on 1 November 2017) and presented in a heat map. The raw spectral data were analyzed with Progenesis QI (Waters Corporation, Milford, MA, USA). After calculating the peak–peak baseline noise, the peak integration, calibration of retention time, and peak normalization, a data matrix of retention time, mass-to-charge ratios, and peak intensity was generated accordingly. The UPLC-MS data matrix was analyzed with the SIMCA-P+14.0 software package (Umetrics, Umeå, Sweden).

Principal component analysis (PCA) was first adopted to discern the metabolic profiles and stability. Then, an orthogonal partial least squares discriminant analysis (OPLS-DA) adopted to supervise the model was applied to extract maximum information from the data matrix and to identify the differential metabolites, which were selected according to the variable important in projection (VIP > 1) and student *t*-test (*p* < 0.05). With the aim of observing which features presented significant variation between girdling and non-girdled leaves, we performed an analysis based on the *p* value and the fold change of the features of our samples. The statistical significance was set to a logarithm of a *p* value < 0.05.

According to the public database, the differential metabolites were identified (http://www.hmdb.ca/, http://www.lipidmaps.org/, accessed on 16 October 2017) with the self-constructed database. The KEGG database (http://www.kegg.jp/kegg/pathway.html, accessed on 20 October 2017) was adopted to annotate the pathways of differential metabolites, which were utilized for further analyses and to screen principle pathways. The principal pathways that mostly corresponded to differential metabolites were observed according to enrichment analysis and hierarchical clustering analysis.

The significance of the difference was analyzed with variance ANOVA or *t-*test with SPSS 8.0 software (v.22, IBM).

## 5. Conclusions

In this study, the metabolic changes in litchi leaves after stem girdling and cold treatment were investigated. The results showed that girdling improved litchi flowering with terminal flushes at the light green stage. Furthermore, the high levels of epinephrine, sucrose, and d-maltose and the low levels of UDP-glucose, d-glucose-6P, d-galactonate, and d-fructose measured in leaves from stem girdled trees during floral induction might be associated with litchi flowering. The stable levels of galactitol, phenylpyruvic acid, acetyl-CoA, linoleic acid, alpha-linolenic acid, 13-HPOT, 9-oxoODE, and 9,12,13-triHOME suggested that girdling could overcome the cooling effect of activating the metabolic pathways of these compounds, which might negatively affect litchi flowering. In conclusion, starch and sucrose metabolism, galactose metabolism, phosphoenolpyruvate metabolism, and linoleic acid metabolism might play essential roles in litchi floral induction. This work contributed to understanding the biological mechanisms behind stem girdling and cold treatment during floral induction.

## Figures and Tables

**Table 1:** Litchi flowering time and quantities in response to girdling and cold temperature treatment.

Treatment	Non-Girdled	Mature	Girdled
Percentage of flowering trees (%)	16.67	100	100
Percentage of flowering shoots (%)	11.08 ± 9.14 b	74.23 ± 17.93 a	71.97 ± 10.06 a
Days to floral initiation	81.50 ± 0.50 a	72.50 ± 2.47 a	78.00 ± 2.07 a
Days to floral differentiation (Male flowers)	29.00 ± 6.00 a	35.00 ± 1.41 a	34.33 ± 2.56 a
Days to floral differentiation (Female flowers)	29.00 ± 4.00 a	33.25 ± 1.03 a	30.00 ± 1.83 a
Panicle length (cm)	10.67 ± 0.16 b	15.52 ± 0.30 a	8.59 ± 0.97 b
Panicle width (cm)	5.04 ± 0.04 b	9.1 ± 0.86 a	6.01 ± 0.84 ab
Panicle length/Panicle width (cm)	2.11 ± 0.01 a	1.86 ± 0.20 ab	1.53 ± 0.09 b

Data values represented mean ± SE (*n* = 6). Different letters in the same row indicate significant differences determined by an unpaired ANOVA at *p* < 0.05.

## Data Availability

The data presented in this study are available in Supplementary Material.

## Supplementary Materials

The following are available online, Figure S1: Orthogonal projections to latent structures-discriminate analysis (OPLS-DA) score plots showed the metabolomics trajectory of picked “Guiwei” litchi leaves in the girdling group and control group under floral induction. An OPLS-DA score plots of all metabolites from six biological replicates of litchi leaves collected at a 0 and 3 days after treatment, respectively. B–F OPLS-DA score plots of all metabolites from six biological replicates of litchi leaves collected at a 3, 4, 23, 63, and 71 days after treatment, respectively. C indicated samples of plants from the control group. T indicated samples of plants from the girdling group, Figure S2: Kyoto Encyclopedia of Genes and Genomes (KEGG) enrichment of metabolites accounting for flowering in “Guiwei” litchi leaves from terminal flush. The size of the circle represents the number of enriched protein molecules. The color indicates the significance of the enrichment at *p*-value, Figure S3: Kyoto Encyclopedia of Genes and Genomes (KEGG) enrichment of metabolites accounting for flowering rate in “Guiwei” litchi leaves from terminal flush. The size of the circle represents the number of enriched protein molecules. The color indicates the significance of the enrichment P-value, Figure S4: Changes in soluble sugars, starch, and Indole-3-acetic acid (IAA) in leaves. Asterisks (*) indicate significant differences determined by an unpaired t-test at *p* < 0.05. Double asterisks (**) indicate significant differences determined by an unpaired *t*-test at *p* < 0.01.

## Sample Availability

Samples of the compounds are not available from the authors.

## Abbreviations

C	Control
DAT	Day after treatment
FDR	False discovery rate
KEGG	Kyoto Encyclopedia of Genes and Genomes
OPLS-DA	Orthogonal projections to latent structures-discriminate analysis
PCA	Principal component analysis
SPAD	Soil and plant analyzer development
T	Girdling treatment
UPLCQ-TOF/MS	Ultra-high performance liquid chromatography-quadrupole time-of-Flight mass spectrometry
VIP	Variable important in projection
