# Supplementary material for: Metabolomics Analysis of Litchi Leaves during Floral Induction Reveals Metabolic Improvement by Stem Girdling
Source: Molecules. 2021 Jul 2;26(13):4048. doi: 10.3390/molecules26134048 (PMC8271987; doi:10.3390/molecules26134048)
Supplement: Supplementary file 1 [file molecules-26-04048-s001.zip › molecules-1243949-SI.pdf]

Supplementary Materials: The following are available online, Figure S1 Orthogonal projections to latent structures-discriminate analysis (OPLS-DA) score plots showed the metabolomics trajectory of picked 'Guiwei' litchi leaves in girdling group and control group under floral induction. A OPLS-DA score plots of all metabolites from six biological replicates of litchi leaves collected at a 0 and 3 days after treatment, respectively. B-F OPLS-DA score plots of all metabolites from six biological replicates of litchi leaves collected at a 3, 4, 23, 63, 71 days after treatment, respectively. C indicated samples of plants from control group. T indicated samples of plants from girdling group. Figure S2 Kyoto Encyclopedia of Genes and Genomes (KEGG) enrichment of metabolites accounting for flowering in 'Guiwei' litchi leaves from terminal flush. The size of the circle represents the number of enriched protein molecules. The color indicates the significance of the enrichment at *P*-value. Figure S3 Kyoto Encyclopedia of Genes and Genomes (KEGG) enrichment of metabolites accounting for flowering rate in 'Guiwei' litchi leaves from terminal flush. The size of the circle represents the number of enriched protein molecules. The color indicates the significance of the enrichment *P*-value. Figure S4 Changes in soluble sugars, starch and Indole-3-acetic acid (IAA) in leaves. Asterisks (*) indicate significant differences determined by an unpaired *t*-test at $p < 0.05$. Double asterisks (**) indicate significant differences determined by an unpaired *t*-test at $p < 0.01$.

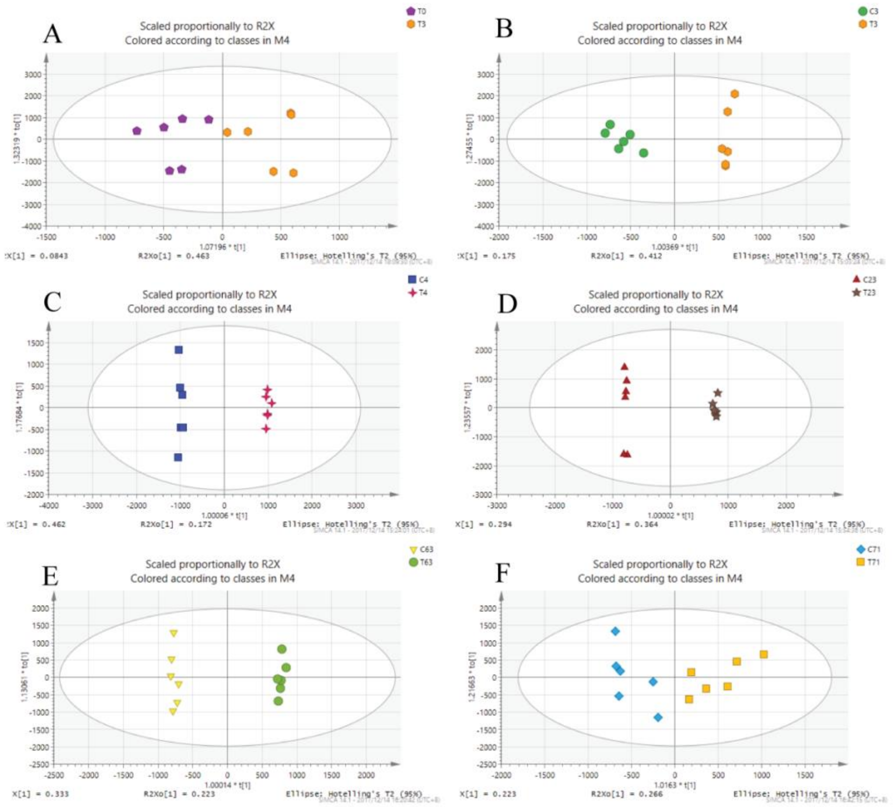

Figure S1

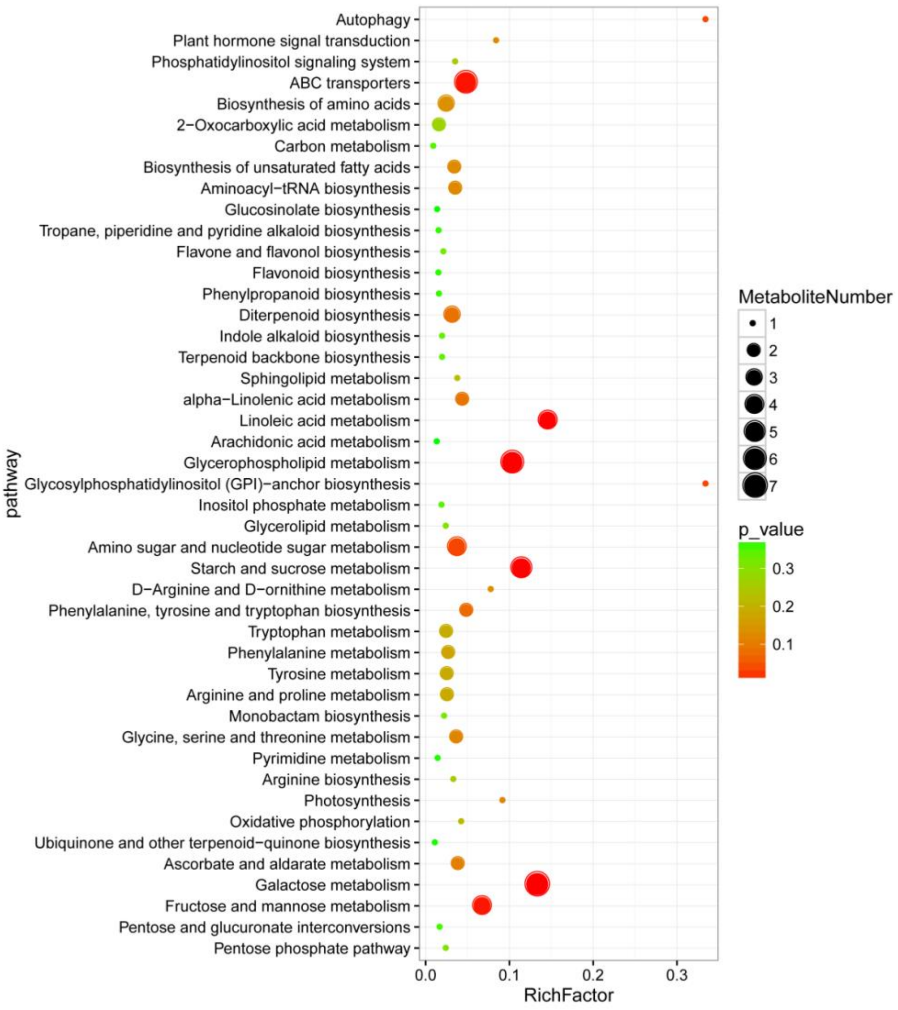
Autophagy
Plant hormone signal transduction
Phosphatidylinositol signaling system
ABC transporters
Biosynthesis of amino acids
2−Oxocarboxylic acid metabolism
Carbon metabolism
Biosynthesis of unsaturated fatty acids
Aminoacyl−tRNA biosynthesis
Glucosinolate biosynthesis
Tropane, piperidine and pyridine alkaloid biosynthesis
Flavone and flavonol biosynthesis
Flavonoid biosynthesis
Phenylpropanoid biosynthesis
Diterpenoid biosynthesis
Indole alkaloid biosynthesis
Terpenoid backbone biosynthesis
Sphingolipid metabolism
alpha−Linolenic acid metabolism
Linoleic acid metabolism
Arachidonic acid metabolism
Glycerophospholipid metabolism
Glycosylphosphatidylinositol (GPI)−anchor biosynthesis
Inositol phosphate metabolism
Glycerolipid metabolism
Amino sugar and nucleotide sugar metabolism
Starch and sucrose metabolism
D−Arginine and D−ornithine metabolism
Phenylalanine, tyrosine and tryptophan biosynthesis
Tryptophan metabolism
Phenylalanine metabolism
Tyrosine metabolism
Arginine and proline metabolism
Monobactam biosynthesis
Glycine, serine and threonine metabolism
Pyrimidine metabolism
Arginine biosynthesis
Photosynthesis
Oxidative phosphorylation
Ubiquinone and other terpenoid−quinone biosynthesis
Ascorbate and aldarate metabolism
Galactose metabolism
Fructose and mannose metabolism
Pentose and glucuronate interconversions
Pentose phosphate pathway
pathway
0.0
0.1
0.2
0.3
RichFactor
MetaboliteNumber
1
2
3
4
5
6
7
p_value
0.3
0.2
0.1

Figure S2

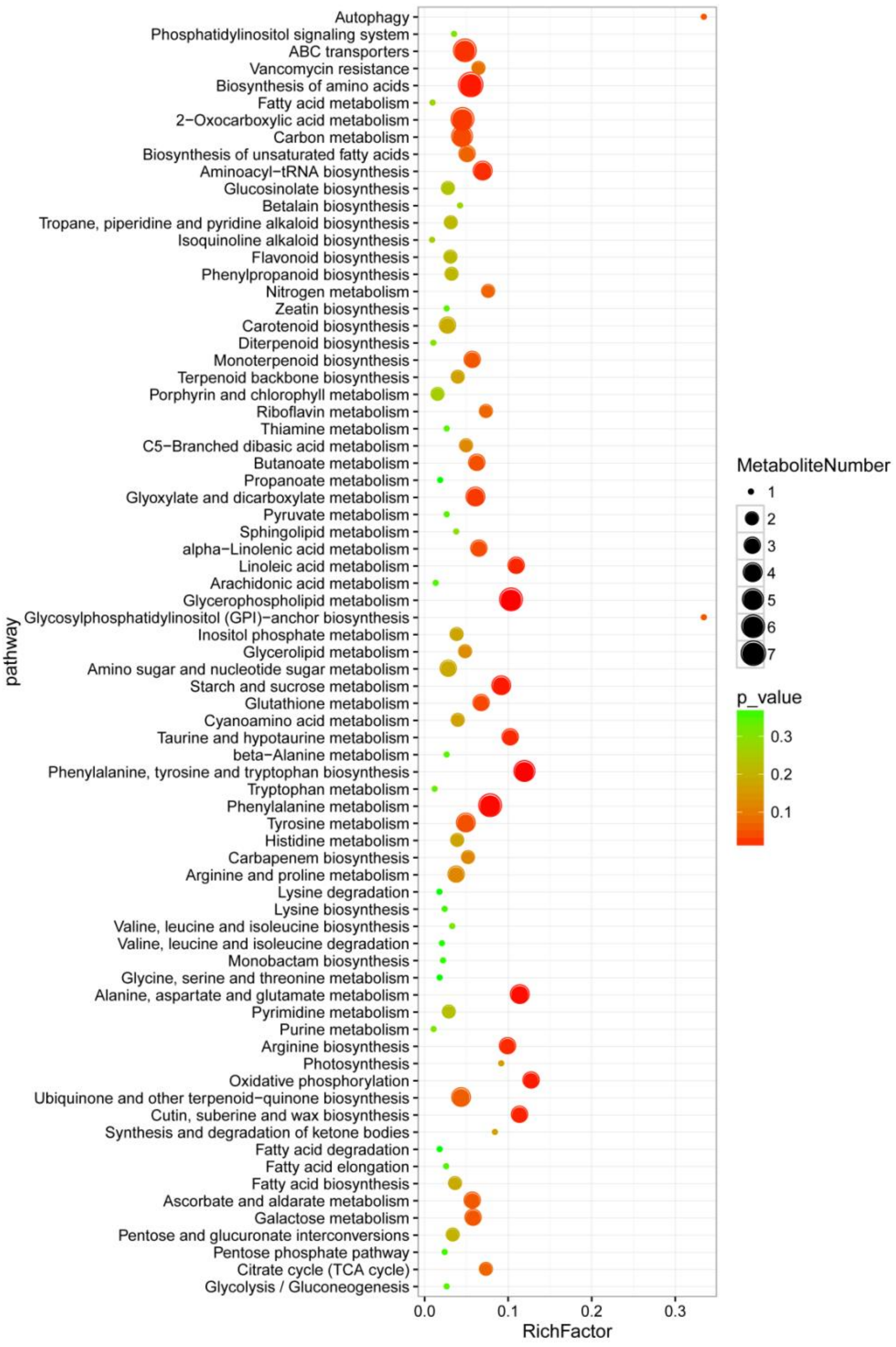
Autophagy
Phosphatidylinositol signaling system
ABC transporters
Vancomycin resistance
Biosynthesis of amino acids
Fatty acid metabolism
2−Oxocarboxylic acid metabolism
Carbon metabolism
Biosynthesis of unsaturated fatty acids
Aminoacyl−tRNA biosynthesis
Glucosinolate biosynthesis
Betalain biosynthesis
Tropane, piperidine and pyridine alkaloid biosynthesis
Isoquinoline alkaloid biosynthesis
Flavonoid biosynthesis
Phenylpropanoid biosynthesis
Nitrogen metabolism
Zeatin biosynthesis
Carotenoid biosynthesis
Diterpenoid biosynthesis
Monoterpenoid biosynthesis
Terpenoid backbone biosynthesis
Porphyrin and chlorophyll metabolism
Riboflavin metabolism
Thiamine metabolism
C5−Branched dibasic acid metabolism
Butanoate metabolism
Propanoate metabolism
Glyoxylate and dicarboxylate metabolism
Pyruvate metabolism
Sphingolipid metabolism
alpha−Linolenic acid metabolism
Linoleic acid metabolism
Arachidonic acid metabolism
Glycerophospholipid metabolism
Glycosylphosphatidylinositol (GPI)−anchor biosynthesis
Inositol phosphate metabolism
Glycerolipid metabolism
Amino sugar and nucleotide sugar metabolism
Starch and sucrose metabolism
Glutathione metabolism
Cyanoamino acid metabolism
Taurine and hypotaurine metabolism
beta−Alanine metabolism
Phenylalanine, tyrosine and tryptophan biosynthesis
Tryptophan metabolism
Phenylalanine metabolism
Tyrosine metabolism
Histidine metabolism
Carbapenem biosynthesis
Arginine and proline metabolism
Lysine degradation
Lysine biosynthesis
Valine, leucine and isoleucine biosynthesis
Valine, leucine and isoleucine degradation
Monobactam biosynthesis
Glycine, serine and threonine metabolism
Alanine, aspartate and glutamate metabolism
Pyrimidine metabolism
Purine metabolism
Arginine biosynthesis
Photosynthesis
Oxidative phosphorylation
Ubiquinone and other terpenoid−quinone biosynthesis
Cutin, suberine and wax biosynthesis
Synthesis and degradation of ketone bodies
Fatty acid degradation
Fatty acid elongation
Fatty acid biosynthesis
Ascorbate and aldarate metabolism
Galactose metabolism
Pentose and glucuronate interconversions
Pentose phosphate pathway
Citrate cycle (TCA cycle)
Glycolysis / Gluconeogenesis
pathway
0.0
0.1
0.2
0.3
RichFactor
MetaboliteNumber
1
2
3
4
5
6
7
p_value
0.3
0.2
0.1

Figure S3

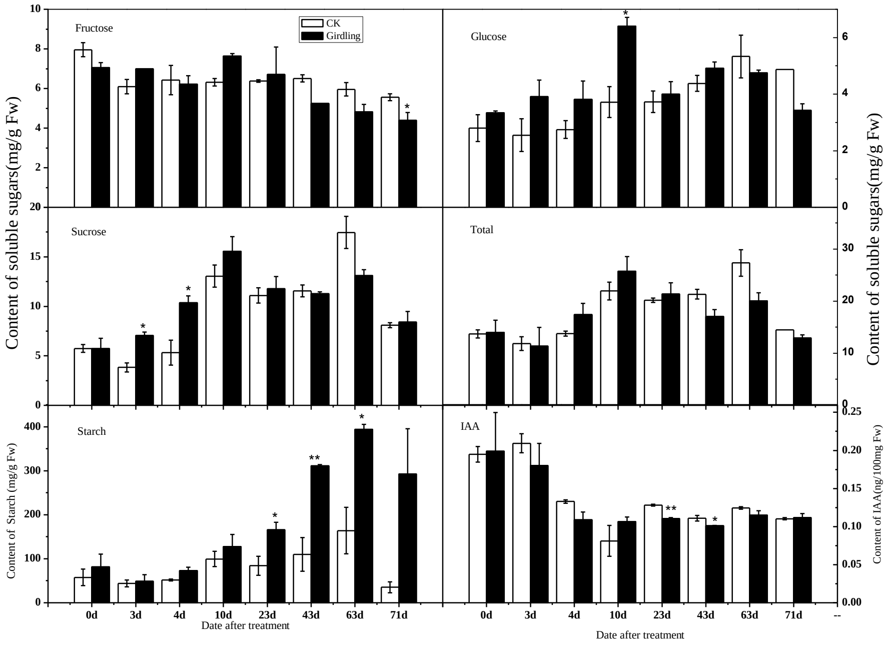

Fructose
CK
Girdling
Glucose
Sucrose
Total
Starch
IAA
Content of soluble sugars(mg/g Fw)
Content of soluble sugars(mg/g Fw)
Content of Starch (mg/g Fw)
Content of IAA(ng/100mg Fw)
0d
3d
4d
10d
23d
43d
63d
71d
Date after treatment
Date after treatment

Figure S4
